# Physical Activity Scaled to Preferred Walking Speed as a Predictor of Walking Difficulty in Older Adults: A 2-Year Follow-up

**DOI:** 10.1093/gerona/glab277

**Published:** 2021-10-11

**Authors:** Laura Karavirta, Heidi Leppä, Timo Rantalainen, Johanna Eronen, Erja Portegijs, Taina Rantanen

**Affiliations:** Faculty of Sport and Health Sciences and Gerontology Research Center, University of Jyväskylä, Finland

**Keywords:** Accelerometer, Cut-point, Disablement, Exercise intensity, Mobility limitation, Physical performance

## Abstract

**Background:**

The usual accelerometry-based measures of physical activity (PA) are dependent on physical performance. We investigated the associations between PA relative to walking performance and the prevalence and incidence of early and advanced walking difficulties compared to generally used measures of PA.

**Methods:**

Perceived walking difficulty was evaluated in 994 community-dwelling participants at baseline (age 75, 80, or 85 years) and 2 years later over 2 km (early difficulty) and 500 m (advanced difficulty). We used a thigh-mounted accelerometer to assess moderate-to-vigorous PA, daily mean acceleration, and relative PA as movement beyond the intensity of preferred walking speed in a 6-minute walking test (PA_rel_). Self-reported PA was assessed using questionnaires.

**Results:**

The prevalence and incidence were 36.2% and 18.9% for early and 22.4% and 14.9% for advanced walking difficulty, respectively. PA_rel_ was lower in participants with prevalent (mean 42 [*SD* 45] vs 69 [91] min/week, *p* < .001) but not incident early walking difficulty (53 [75] vs 72 [96] min/week, *p* = .15) compared to those without difficulty. The associations between absolute measures of PA and incident walking difficulty were attenuated when adjusted for preferred walking speed.

**Conclusions:**

The variation in habitual PA may not explain the differences in the development of new walking difficulty. Differences in physical performance explain a meaningful part of the association of PA with incident walking difficulty. Scaling of accelerometry to preferred walking speed demonstrated independence on physical performance and warrants future study as a promising indicator of PA in observational studies among older adults.

Walking performance is the key element of mobility that enables continuation of independent living in aging (1). Walking difficulty over a longer distance, such as 2 km, is an early sign of decline in functional ability and precedes further disability and dependence (2,3), while difficulty in the shorter distance represents a critical level under which independent mobility may become threatened (4). Walking performance is determined by an interplay of neuromuscular, cardiorespiratory, and sensory function which all decrease in aging (5). Habitual physical activity (PA) may help slow down the decline in the determinants of walking performance and help in maintaining walking ability and independence.

In older adults, walking forms a large portion of daily aerobic PA, which can be assessed in large-scale studies using accelerometry technology. Previous studies have shown that persons with walking difficulty accumulate less accelerometry-based PA compared to people without walking difficulty (6,7). This association is often considered as an indication of the health-enhancing effect of PA. Yet, people cannot freely modify their volume and intensity of PA but instead, they can be active within the limits of their physical capacity. In older age, maximal exercise capacity declines and approaches the level required for daily tasks, which makes performing the tasks more strenuous (8,9). Older adults may continue their daily tasks but, for example, reduce their walking speed to compensate for the decline in physical capacity (2), which leads to lower absolute acceleration in the accelerometry surveillance. Accelerometry-based assessment of moderate-to-vigorous PA (MVPA) relies heavily on exceeding a universal intensity cut-point, which may be unattainable for people with low physical performance (10). Thus, physical performance underlying the baseline level of PA may strongly contribute to the association between accelerometry-based PA and walking difficulty (11).

We have recently developed an accelerometry-based relative measure of PA (PA_rel_) that takes individual variation in walking performance into account (12). The computation of PA_rel_ uses preferred walking speed as a cut-point, which is considered as the lower limit for challenging the body beyond the usual daily stimuli, that is, exercise intensity that a person is accustomed to in daily life. Exceeding the usual intensity and volume of exercise is the foundation of physical adaptation (13). PA_rel_ is quantified as the total amount of accelerometry-based, free-living PA per week that equals or exceeds the intensity level of preferred walking speed that we assessed in a modified 6-minute walking test (6MWT) (12). Preferred walking speed stays relatively stable throughout adult life until the age of 65, after which it is more strongly determined by increased energetic cost of walking and declined physical performance (14–16). PA_rel_ is based on the same principle as individually tailored PA interventions and PA guidelines in that it considers intensity relative to individual performance level. Our previous study showed that PA_rel_ was not associated with walking speed, age, or sex (12). Therefore, PA_rel_ provides an accelerometry-based measure of PA that can be used to study the independent role of free-living daily PA in the prevalence and incidence of walking difficulty in older people.

Recently, we demonstrated an association between prevalent walking difficulty and accelerometer-based measures of PA such as intensity and amount of walking in free-living (7). This study extends the previous findings by investigating the incidence of walking difficulty and including a novel relative measure of PA (PA_rel_) that is independent of the prevailing walking performance. The aim of the study was to investigate the associations between PA_rel_ and the prevalence and incidence of early and advanced walking difficulties compared to the associations of several other accelerometry-based and self-reported measures of PA with the prevalence and incidence of walking difficulties. We hypothesize that the associations are weaker when the effect of physical performance is controlled for.

## Method

### Participants and Study Design

We present cross-sectional and longitudinal results of the observational “Active aging—resilience and external support as modifiers of the disablement outcome” (AGNES) study. The baseline data were collected between September 2017 and December 2018, and the baseline study protocol has been previously reported in detail (17). In brief, our sample comprises 3 age cohorts of older men and women (75, 80, and 85 years) living independently in the city of Jyväskylä in Central Finland. We excluded people who were unable to communicate. At baseline, the participants completed a postal questionnaire, home interview, PA surveillance for 3–7 days, and assessments in the research center.

The AGNES-coronavirus disease 2019 (COVID-19) survey, which we conducted in May–June 2020 during the COVID-19 pandemic, provided us an opportunity to collect follow-up data on walking difficulty for the current prospective analyses. A series of restrictions and recommendations were issued in Finland in March 2020. Special attention was paid to the safety of older people, who were considered to be at the highest risk for developing a serious form of the disease. Thus, social distancing, that is, limiting close contact and avoiding places with other people, was recommended especially for people older than 70 years old. Activity destinations such as restaurants, activity centers, and gyms were closed (18). Although curfew was not imposed in Finland, moving outdoors may have declined.

A flow chart of the follow-up study has been previously published (19). Of the 1 021 baseline participants, 985 were surviving and had not withdrawn their consent and formed the target group for the follow-up data collection. Data were received from 809 participants using postal questionnaires (*n* = 802) or in case of difficulty or unwillingness to fill in the questionnaire, by interviews over the phone (*n* = 7). At baseline, 27 participants had missing data on self-reported early and 27 participants on advanced walking difficulty. There were 634 (and 771) participants without early (and advanced) walking difficulty at baseline, respectively, of which 12 (17) were deceased by the follow-up, 86 (110) did not respond, 6 (7) were unable to respond, and 1 (1) had moved to a care home. In addition, there were 4 participants without data on perceived walking difficulty in both 2 km and 500 m during the follow-up. The remaining 525 participants without early or advanced difficulties at baseline and 632 participants without advanced difficulties were included in the incidence analysis of early and advanced walking difficulties, respectively.

Anthropometric and physical performance measures are available for those who participated in the measurements in the research center at baseline (*n* = 895). Self-reported PA measures were available for 994 participants. Approximately half of the original sample participated in the accelerometry surveillance. Those who participated in the surveillance did not differ from the people who only participated in the research center assessments in terms of sex, self-rated health, or advanced walking difficulty, but had higher self-reported PA and higher walking speed (20).

The ethical committee of the Central Finland Hospital district provided an ethical statement about AGNES on August 23, 2017 and May 13, 2020. Prior to the assessments, participants signed an informed consent and they were allowed to withdraw their consent at any time during the study. The study follows the principles of the Declaration of Helsinki.

### Outcome Variables

*Walking difficulty* was evaluated as *early and advanced* over distances of 2 km and 500 m, respectively. Perceived difficulty was assessed using a standardized question in a face-to-face interview (baseline) and in a postal questionnaire (follow-up): “Do you have difficulty in walking 2 km/500 m?” (2). The response options were (a) “able to manage without difficulty,” (b) “able to manage with some difficulty,” (c) “able to manage with a great deal of difficulty,” (d) “able to manage only with help of another person,” and (e) “unable to manage even with help.” Two categories for early and advanced walking difficulty were created: (1) no difficulty (response option a) and (2) difficulty (response options b–e).

### Independent Variables

*PA* was assessed using 3 methods: Yale Physical Activity Survey (YPAS) for older adults (21), a modified version of a single question on the self-reported level of PA (22), and accelerometry (12). In the YPAS questionnaire, participants were asked how many times and for how long at a time they performed PA of different intensities during the past month. A subscore for each intensity level was computed as the product of frequency and duration. The YPAS total score was computed by giving different weights for each intensity subscore: 5 for vigorous, 4 for leisure walking, 3 for moving about, 2 for standing, and 1 for sitting and summing the weighted subscores (range 0–137). Higher scores indicate a higher total volume of PA. Finally, the total score is adjusted with the participants estimate on whether their PA in the previous month differed from their PA in other annual seasons (winter, spring, summer, autumn) on a 5-point scale from 1.3 (lot more) to 0.7 (lot less) (21). We additionally estimated weekly minutes of walking and vigorous PA. The responses on frequency were recoded to 0 (not at all), 1 (1–3 times per month), 2 (1–2 times per week), 4 (3–5 times per week), and 6 (5+ times per week). The responses on duration were recoded to 20 (10–30 min), 40 (30–50 min), and 60 (60+ min). Minutes per week at each intensity zone were calculated as the product of the frequency and duration code (20).

In the single question on self-reported PA level, the participants were asked to choose the description that reflects their level of PA over the last year: (a) hardly any activity, mostly sitting, (b) light PA, such as light household tasks, (c) moderate PA about 3 hours a week: walking longer distances, cycling, and domestic work, (d) moderate PA at least 4 hours a week or heavier PA 1–2 hours a week, (e) heavier PA or moderate exercise for at least 3 hours a week, and (f) competitive sports (22,23).

The accelerometry data collection and numerical analyses have been described in detail previously (12). Briefly, a thigh-worn accelerometer (triaxial, sampling continuously at 100 Hz, 13-bit ±16 g, UKK RM42; UKK Terveyspalvelut Oy, Tampere, Finland) was taped onto the anterior aspect of the mid-thigh of the dominant leg by a research assistant. The participants were asked to wear the monitor for a minimum of 7 consecutive days and to keep a simple log on their exercise sessions and possible nonwear periods. High-pass filtered vector magnitude (HPFVM, in g) was calculated from the raw accelerometry records in 5-second nonoverlapping epochs after applying autocalibration following the procedure described by White et al. (24). The calibrations for each particular accelerometer were pooled, sorted based on the vector magnitude of the calibration coefficients, and the middle-most (rounded down if even number of calibrations were obtained) calibration was utilized for all files measured with the accelerometer.

For the analysis of the relative intensity cut-point, a modified 6MWT was performed in the research center while wearing the accelerometer. The test was performed in an indoor 20-m corridor at a preferred pace. The mean HPFVM of the 5-second epochs of the 6MWT was recorded as the outcome. Continuous walking throughout the test was required for the analysis of mean 6MWT acceleration (12).

For PA analyses, the whole series of 5-second epochs were split into full 24-hour days from midnight to midnight. Three values were produced as outcomes for each day:

(1) Daily average acceleration: the mean HPFVM (in mg) of all of the recorded 5-second epochs (25),
(2) Absolute MVPA: the number of epochs at or above the acceleration that corresponds to 3 METs based on White et al.’s linear equation for thigh-measured HPFVM (24). For this, we required 2 activity-induced METs plus one from resting metabolism, which resulted in HPFVM ≥0.24 g.
(3) Relative PA: the number of epochs above or equal to the mean acceleration calculated during the 6MWT (12).

We only included days with complete data without any nonwear, which was visually verified from the data. Only participants with at least 3 successfully recorded days were included in the subsequent analyses. The mean of the days scaled for a full week was used as the outcome for each participant.

### Covariates

Age, sex, number of self-reported physician-diagnosed chronic conditions, self-reported years of education, and walking speed in the 6MWT were selected as potential covariates. The total number of physician-diagnosed chronic conditions was calculated from a list of conditions, which were self-reported during the home interview. The list included 34 items and an open-ended question about any other physician-diagnosed chronic conditions. Age and sex of the participants were derived from the Population Information System in the context of recruitment. Self-reported number of years of education was used as an indicator of socioeconomic status. Physical assessments in the research center included standard objective anthropometric measurements of height and body mass. Body mass index was calculated dividing body mass in kilograms by height squared in meters (kg/m^2^). As previously mentioned, 6MWT was performed at a preferred pace. Total distance walked by the participants was measured, and average speed was calculated representing preferred walking speed (12).

### Participant Characteristics

Lower-extremity physical performance was assessed in the participant’s home using the Short Physical Performance Battery (SPPB) (26). The battery comprises tests on standing balance, walking speed over a 3-m distance, and the ability to rise from a chair. A sum score was calculated (range 0–12), where higher scores indicate better performance. Maximal isometric knee extension strength was measured in the research center in a sitting position using an adjustable dynamometer chair (Metitur Ltd, Jyväskylä, Finland) (27).

As the follow-up was conducted during the COVID-19 pandemic, we additionally evaluated self-reported change in PA due to the restrictions and social-distancing recommendations. For this purpose, a single question was adapted from the seasonal adjustment score of the YPAS (21): “Have you changed your physical activity/exercise habits during the COVID-19 restrictions?” The response options were (a) “No,” (b) “I am a lot more active,” (c) “I am a little more active,” (d) “I am a little less active,” and (e) “I am a lot less active.”

### Statistical Analyses

Participant characteristics are reported in percentages for categorical variables and means with standard deviations for continuous variables, stratified for the prevalence and incidence of walking difficulty. Differences between the groups were tested with chi-square tests, independent *t*-tests, or Mann–Whitney *U* tests. Correlations between PA and preferred walking speed were tested with Pearson’s correlation coefficient. Logistic regression with actual PA units and *z*-scores (Supplementary Methods) was used to test the crude association between PA and the incidence of walking difficulty (Model 1, unadjusted). Age, sex, number of chronic conditions, and years of education at baseline were used as covariates in the adjusted model (Model 2). The fully adjusted Model 3 additionally includes preferred walking speed in 6MWT as a covariate for all predictors except for PA_rel_, which is already scaled to preferred walking speed. It is well known that regular PA can improve walking speed in older people and thus be on the causal pathway between PA and incident walking difficulty. However, because walking speed was assessed at approximately the same time as PA and the current measures of PA mainly catch the most recent level of PA, we believe that in this case, walking speed is not on the causal pathway and should be considered as a potential modifier or confounder of the relationship between PA and walking difficulty. Moderation was tested as the PA × walking speed interaction using logistic regression. Statistical significance was set at *p* < .05.

## Results

### Prevalence of Early and Advanced Walking Difficulty

Of the 994 participants, 36.2% and 22.4% reported early or advanced walking difficulty at baseline, that is, difficulty over 2 km or 500 m distance, respectively. The prevalence was larger in women in terms of early walking difficulty (Table 1), but similar between men and women (*p* = .30) in terms of advanced difficulty (Supplementary Table 1). Number of chronic conditions and BMI were higher, and physical performance was lower among those who perceived early or advanced difficulty.

**Table 1.** Participant Characteristics at Baseline Stratified for the Prevalence and Incidence of Early (2 km) Walking Difficulty

	Prevalence (baseline)			Incidence (2-year follow-up)		
	No Difficulty (*n* = 634)	Prevalent Difficulty (*n* = 360)		No Difficulty (*n* = 426)	Incident Difficulty (*n* = 99)	
	% (*n*)/Mean (SD)	% (*n*)/Mean (SD)	*p* ^a^	% (*n*)/Mean (SD)	% (*n*)/Mean (SD)	*p* ^a^
Age (years)			<.001			.003
75	73.6 (332)	26.4 (119)		85.3 (244)	14.7 (42)	
80	62.5 (203)	37.5 (122)		79.9 (131)	20.1 (33)	
85	45.4 (99)	54.6 (119)		68.0 (51)	32.0 (24)	
Sex			<.001			.35
Men	70.4 (297)	29.6 (125)		79.4 (193)	20.6 (50)	
Women	58.9 (337)	41.1 (235)		82.6 (233)	17.4 (49)	
			*p* ^**b**^			*p* ^b^
Chronic conditions (count)	2.8 (1.7)	4.4 (2.2)	<.001	2.7 (1.7)	3.4 (1.8)	<.001
Education (years)	11.8 (4.3)	11.0 (4.1)	.003	12.4 (4.4)	10.6 (3.7)	<.001
SPPB score (0–12)	10.7 (1.5)	8.4 (2.9)	<.001	11.0 (1.2)	10.1 (1.9)	<.001
Height (m)	(*n* = 594)	(*n* = 301)	*p* ^c^	(*n* = 410)	(*n* = 87)	*p* ^**c**^
Men	1.72 (0.06)	1.71 (0.06)	.178	1.73 (0.06)	1.72 (0.07)	.258
Women	1.59 (0.05)	1.57 (0.05)	.002	1.59 (0.05)	1.60 (0.05)	.129
Body mass (kg)						
Men	78.3 (11.1)	83.0 (15.4)	.005	78.0 (10.9)	83.5 (11.1)	.004
Women	67.5 (10.9)	74.1 (12.6)	<.001	66.5 (10.6)	73.2 (11.3)	.001
BMI (kg/m^2^)						
Men	26.4 (3.5)	28.3 (4.9)	<.001	26.1 (3.3)	28.4 (3.5)	<.001
Women	26.7 (3.9)	30.0 (5.1)	<.001	26.4 (3.9)	28.5 (4.0)	.002
6MWT speed (m/s)	(*n* = 588)	(*n* = 265)		(*n* = 406)	(*n* = 87)	
Men	1.27 (0.18)	0.97 (0.24)	<.001	1.29 (0.17)	1.18 (0.17)	<.001
Women	1.20 (0.19)	0.96 (0.21)	<.001	1.25 (0.17)	1.05 (0.20)	<.001
Knee extension strength (N)	(*n* = 591)	(*n* = 294)		(*n* = 408)	(*n* = 87)	
Men	435 (101)	371 (97)	<.001	446 (97)	414 (98)	.060
Women	300 (78)	256 (81)	<.001	311 (77)	267 (77)	.001

*Note:* SPPB = Short Physical Performance Battery; BMI = body mass index; 6MWT = 6-minute walking test at preferred speed. *p* values are calculated with ^a^Chi-square test, ^b^Mann–Whitney *U* Test, or ^c^Independent *t*-test.

### Incidence of Early and Advanced Walking Difficulty

The incidence of early walking difficulty was 18.9% in 2 years. Altogether 14.9% of participants developed new advanced walking difficulty of which 50.0% had early walking difficulty at baseline. The incidence of early (Table 1) and advanced (Supplementary Table 1) walking difficulty was larger in the older age groups but similar in men and women. Differences between the incidence groups were very similar in terms of early and advanced walking difficulties, except for the years of education which was not significant for advanced difficulty (Supplementary Table 1, *p* = .57).

### Associations Between Walking Difficulty and PA

Participants with prevalent early (Table 2) or advanced (Supplementary Table 2) walking difficulty had lower PA in all self-reported and accelerometry-based measures compared to participants with no difficulty. The differences were also significant between the incidence groups, except for PA_rel_, that is, PA above the intensity corresponding to preferred walking speed, which was not significantly different between the groups, either in terms of early or advanced difficulty.

**Table 2.** Physical Activity From the Self-Report and Accelerometry Surveillance at Baseline Stratified for Prevalence and Incidence of Early (2 km) Walking Difficulty

	Prevalence (baseline)			Incidence (2-year follow-up)		
	No Difficulty	Prevalent Difficulty		No Difficulty	Incident Difficulty	
	Mean (*SD*)	Mean (*SD*)	p	Mean (*SD*)	Mean (*SD*)	p
Self-report	(*n* = 634)	(*n* = 360)		(*n* = 426)	(*n* = 99)	
YPAS total score	63 (22)	43 (20)	<.001	65 (22)	55 (20)	<.001
YPAS vig + walk (min/week)	286 (142)	168 (120)	<.001	300 (141)	239 (131)	<.001
YPAS vig (min/week)	126 (101)	64 (73)	<.001	133 (100)	99 (93)	.001
YPAS walk (min/week)	160 (97)	104 (82)	<.001	166 (97)	140 (93)	.014
PA level (1–6)	3.8 (0.8)	2.9 (0.9)	<.001	4.0 (0.7)	3.5 (0.8)	<.001
Accelerometry surveillance	(*n* = 344)	(*n* = 142)		(*n* = 248)	(*n* = 45)	
Average acceleration (mg)	26.3 (8.1)	18.7 (6.3)	<.001	27.1 (8.3)	22.4 (6.2)	<.001
MVPA_abs_ (min/week)	258 (165)	117 (108)	<.001	275 (170)	178 (128)	<.001
PA_rel_ (min/week)	69 (91)	42 (45)	<.001	72 (96)	53 (75)	.147

*Notes:* YPAS = Yale Physical Activity Survey; vig = vigorous; PA = physical activity; MVPA_abs_ = moderate-to-vigorous PA based on an absolute accelerometry cut-point; PA_rel_ = PA relative to preferred walking speed. *p* values are calculated with an Independent *t*-test.

Results of the logistic regression analysis regarding the incidence of advanced and early difficulty were similar. The results on early difficulty were chosen to be presented over advanced difficulty due to the small number of participants with incident advanced difficulty and accelerometry data (*n* = 38). The analysis showed that lower self-reported PA and accelerometry-based absolute PA but not PA_rel_ were associated with incident early walking difficulty (Table 3, Model 1).

**Table 3.** Logistic Regression for the Incidence of Early (2 km) Walking Difficulty in the 2-Year Follow-up

		Model 1 Unadjusted		Model 2 Adjusted		Model 3 Fully Adjusted	
		OR	95% CI	OR	95% CI	OR	95% CI
*Self-report*							
YPAS total score (10 units)	*n* = 486	0.80*	0.71–0.90	0.81*	0.72–0.92	0.86*	0.76–0.98
YPAS vig + walk (10 min/day)	*n* = 487	0.79*	0.69–0.90	0.80*	0.70–0.92	0.85*	0.74–0.98
YPAS vig (10 min/day)	*n* = 489	0.79*	0.66–0.95	0.80*	0.66–0.96	0.89	0.74–1.08
YPAS walk (10 min/day)	*n* = 489	0.79*	0.65–0.95	0.79*	0.65–0.96	0.81*	0.66–0.99
PA level (1–6)	*n* = 492	0.44*	0.32–0.61	0.46*	0.33–0.64	0.60*	0.42–0.85
*Accelerometer surveillance*							
Average acceleration (10 mg)	*n* = 291	0.40*	0.24–0.68	0.42*	0.24–0.74	0.62	0.36–1.09
MVPA_abs_ (10 min/day)	*n* = 291	0.70*	0.57–0.86	0.71*	0.58–0.88	0.82	0.67–1.00
PA_rel_ (10 min/day)	*n* = 281	0.84	0.61–1.14	0.81	0.58–1.12	—	—

*Notes:* OR = odds ratio; CI = confidence interval; YPAS = Yale Physical Activity Survey; vig = vigorous; PA = physical activity; MVPA_abs_ = moderate-to-vigorous PA based on absolute accelerometry cut-point; PA_rel_ =PA relative to preferred walking speed. Model 2 is adjusted for age, sex, number of chronic conditions, and years of education. Model 3 is additionally adjusted for preferred walking speed in a 6-minute walking test.

*Statistical significance (*p* < .05).

After adjusting for age, sex, number of chronic conditions, and years of education, the same associations remained significant (Table 3 and Supplementary Table 3, Model 2). Preferred walking speed in 6MWT was positively associated with all measures of PA (*r* = 0.24–0.35, *p* < .001) except for YPAS walking time (*r* = 0.09, *p* = .055) and PA_rel_, where the association was negative (*r* = −0.20, *p* = .001). Preferred walking speed × PA interaction was not significant for any of the PA measures studied (*p* = .30–.95). Therefore, we included preferred walking speed as a confounder in Model 3. After the adjustment with preferred walking speed, the associations of accelerometry-based PA with the incidence of walking difficulty were attenuated. For all self-reported measures of PA except for vigorous PA, the associations remained statistically significant (Table 3, Model 3).

### Sensitivity Analyses

To test the robustness of our findings, we stratified the logistic regression analyses based on SPPB scores (≤10, *n* = 165 vs ≥11, *n* = 360). The associations regarding accelerometry did not change materially. The associations of self-reported PA with incident walking difficulty were attenuated but were similar in participants with both high and low SPPB scores.

## Discussion

This study produced new information on the associations between habitual PA and walking difficulty in older adults. PA_rel_ that was individually scaled using preferred walking speed and thus, independent of walking performance at baseline, was lower in older adults with prevalent walking difficulty compared to those who perceived no difficulty. This difference was not observed in terms of the incidence of early or advanced difficulty. Furthermore, the association between accelerometry-based MVPA and incident walking difficulty was attenuated after adjusting the analyses for preferred walking speed. The majority of self-reported measures of PA contributed significantly to incident walking difficulty, regardless of the adjustment. These results suggest that baseline walking performance is a significant factor underlying the association between absolute accelerometry-based measures of PA and incident walking difficulty in older adults. This finding encourages the use of PA assessment methodology that takes baseline physical performance into account when investigating future development of walking difficulty and other mobility limitations.

There are at least 2 advantages in using relative and self-reported measures over absolute accelerometry-based measures of PA when assessing the independent role of PA in the incidence of walking difficulty. First, relative accelerometry-based and self-reported measures of PA take individual physical performance level into account. When an individual is asked to recall the number of times and average duration of PA on a usual week, there is no requirement for exceeding a certain intensity level. Similarly, the rationale behind PA_rel_ was to assess PA independent of physical performance by scaling accelerometry to individual preferred walking speed. In contrast, accelerometer-based assessment of MVPA is based on an absolute intensity cut-point which may exceed individual physical capacity. Averaging the accelerometer signal over a whole day is similarly influenced by individual ability to maintain a given PA intensity (12). Thus, in older adults, accelerometry-based absolute measures of PA may be largely determined by physical performance and in a smaller degree by freely regulated physical behavior (10). Second, self-reported measures of PA can include activities that improve muscular strength and balance. In the LIFE study, for example, the intervention that had a significant impact on the ability to walk 400 m included several components, such as aerobic, resistance, and flexibility training (28). Of these, only aerobic training can be captured with accelerometers with reasonable accuracy, and only approximately 20% of the intervention-induced change in self-reported activity minutes was observed in the accelerometry-based MVPA (29).

Challenges related to using absolute values of acceleration as the measure of total PA or as a universal cut-point for MVPA have been widely acknowledged, especially when assessing PA in older people (10,30–32). Older adults have lower cardiorespiratory fitness (9), lower resting metabolic rate, that is, one metabolic equivalent (MET) (32), slower gait speed, and higher energy expenditure per distance walked (14) than younger adults who question the validity of the universal 3 MET cut-points in the assessment of MVPA. Self-reported physical fitness is associated with the number of steps, MVPA, daily mean MET, and time spent sedentary in older adults (33). In epidemiological studies, physical performance is rarely acknowledged as a confounder in the association between accelerometer-based MVPA and mobility disability. Recently though, mobility (ability to walk a quarter mile or climb 10 stairs) as a component of health has been investigated as a confounder in accelerometer–mortality relationship. The findings indicated a larger risk reduction for all-cause mortality using accelerometers compared to self-reported PA, which may be due to reversed causality, that is, the impact of physical performance on the baseline PA. PA–mortality associations may be overestimated if statistical adjustment for health is limited, especially when investigating older age groups (34). Based on the present findings, a similar risk for overestimation exists in the PA–walking ability relationship, especially when using absolute PA measures based on accelerometry. We want to point out the bidirectional relationship between physical performance and PA, as walking speed can be affected by a physically active lifestyle and thus mediate the association between PA and walking difficulty. However, walking speed measured at the same time as PA can be safely considered as a confounder, as has also been previously suggested (34).

It seems that the volume of habitual PA_rel_ observed in this study was not high enough to slow down the development of new walking difficulty. However, we cannot rule out that the short follow-up contributes to the lack of prospective associations. In the cross-sectional analyses, people who perceived no prevalent walking difficulty accumulated a larger amount of PA_rel_ than people with early or advanced difficulty. It seems that a person without difficulty accumulates more PA than a person who perceives difficulty, irrespective of walking speed. Walking speed does not assess the individual perception of walking ability. Perceived difficulty may stem from pain, fear, or involuntary modifications to customary performance (35). Therefore, the experience of difficulty may lead to further decline in PA. Intervention studies aiming to enhance the walking performance of older adults often include exercises targeted to improve muscle strength and balance, and they have shown promise in slowing down the progression of walking difficulty (28,36). Dose–response relationship between MVPA and the onset of disability to walk 400 m has been previously shown using accelerometer counts, but because the participants also performed weight training, it is not possible to conclude if the increased amount of walking or weight training or both combined induced the delayed onset of disability (29). The dose–response relationship of aerobic PA and incident walking difficulty remains unknown.

The associations between PA and walking difficulty were similar in terms of early and advanced difficulties. The main differences were the expected higher incidence of early compared to advanced difficulty and the higher prevalence of walking difficulty in women compared to men in terms of early walking difficulty only. Only half of the present participants who developed new advanced walking difficulty perceived early difficulty at baseline, which shows that walking difficulty may develop quite rapidly. Although the follow-up measurements coincided with the early months of the COVID-19 pandemic, the present 2-year incidence of walking difficulty (19% in 2 km and 15% in 500 m) was not exceptionally high compared to previous studies on similar populations and the same follow-up duration: 31% in 2 km (37) and 16% in 500 m (38) in community-dwelling people of similar age and physical performance. Our additional analysis indicated that 35.2% of the present participants reported reduced habitual PA due to the pandemic restrictions, whereas others reported no change (46.2%) or an increase (18.6%). Thus, it seems that 2–3 months of pandemic restrictions did not accelerate the development of walking difficulty, but it may increase the risk of new mobility limitations if continued. Decreased life-space mobility in the same participants may indicate an increased risk of developing disability in short term (19). Because the activity destinations remain partly closed and recommendations for social distancing continue a year later, it will be important to evaluate regularly their effect on the development of mobility disability of older people.

There are both strengths and limitations in this study. We were able to use the baseline data of a relatively large population-based sample of older adults from 3 age cohorts. We used an exceptionally large selection of different PA assessment methods including a novel relative PA index which enabled the investigation of the independent role of PA but without the obvious limitation of PA recollection as in the self-report. During the follow-up, there was a high response rate and few missing responses. As for the limitations, we had to rely on postal questionnaires during the follow-up data collection as all research requiring physical contact with participants was discontinued in our university due to the COVID-19 pandemic. Therefore, we cannot rule out a potential bias due to the difference in the data collection method between the baseline (home interview) and the COVID-19 survey (postal questionnaire). We were also not able to repeat the accelerometry surveillance, which might have revealed changes in activity behavior more objectively than the questionnaire. The present participants formed a population-based sample, but the sample size was smaller and slightly better functioning in the accelerometry-based compared to self-reported metrics (20), which could result in type I error of rejecting a true null hypothesis. The incidence of walking difficulty may have been larger in the whole population, which may attenuate the associations reported here. However, the physical performance level of the present participants at baseline was comparable to a previous study in terms of SPPB (37). Finally, using 24-hour accelerometry may result in higher PA, especially higher average acceleration, compared to recording awake time only. To date, the low accuracy of accelerometry in distinguishing lying awake from sleep does not allow their separate analysis (39).

To conclude, this study showed that differences in physical performance explain a meaningful part of the association of PA with incident walking difficulty. Therefore, the variation in habitual PA assessed relative to physical performance did not explain the differences in the development of new walking difficulty. The use of absolute accelerometry-based measures of PA may overestimate the association between PA and the incidence of walking difficulty as these were not independent of the baseline physical performance level. Self-reported measures of PA seem more robust to the effect of physical performance in older adults compared to absolute accelerometry-based PA, potentially due to the subjectively rated intensity of activity instead of a universal absolute cut-point. Scaling of accelerometry to preferred walking speed demonstrated independence on physical performance and warrants future study as a novel indicator of PA in observational studies among older adults.

## Supplementary Material

glab277_suppl_Supplementary_Materials
