# Supplementary material for: Physical Activity Scaled to Preferred Walking Speed as a Predictor of Walking Difficulty in Older Adults: A 2-Year Follow-up
Source: J Gerontol A Biol Sci Med Sci. 2021 Oct 11;77(3):597–604. doi: 10.1093/gerona/glab277 (PMC8893185; doi:10.1093/gerona/glab277)
Supplement: glab277_suppl_Supplementary_Materials [file glab277_suppl_supplementary_materials.pdf]

Physical activity scaled to preferred walking speed as a predictor of walking difficulty in older adults: a 2-year follow-up.

Online-Only Supplemental Material

eTable 1. Participant characteristics at baseline stratified for the prevalence and incidence of advanced (500 m) walking difficulty.

eTable 2. Physical activity from the self-report and accelerometry surveillance at baseline stratified for the prevalence and incidence of advanced (500 m) walking difficulty.

eMethods. Logistic regression analysis using z-scores.

eTable 3. Logistic regression for the incidence of early (2 km) walking difficulty in the 2-year follow-up using z-scores for physical activity.

eTable 1. Participant characteristics at baseline stratified for the prevalence and incidence of advanced (500 m) walking difficulty.

	Prevalence (baseline)			Incidence (2-yr follow-up)		
	**No difficulty** (n=771)	**Prevalent difficulty** (n=223)		**No difficulty** (n=538)	**Incident difficulty** (n=94)	
	**% (n)**	**% (n)**	**p [a]**	**% (n)**	**% (n)**	**p [a]**
Age (years)			<0.001			0.041
75	84.3 (380)	15.7 (71)		88.6 (287)	11.4 (37)	
80	78.8 (256)	21.2 (69)		82.0 (169)	18.0 (37)	
85	61.9 (135)	38.1 (83)		80.4 (82)	19.6 (20)	
Sex			0.305			0.975
Men	79.1 (334)	20.9 (88)		85.1 (228)	14.9 (40)	
Women	76.4 (437)	23.6 (135)		85.2 (310)	14.8 (54)	
	**Mean (SD)**	**Mean (SD)**	**p [b]**	**Mean (SD)**	**Mean (SD)**	**p [b]**
Chronic conditions (count)	3.0 (1.8)	4.7 (2.3)	<0.001	2.8 (1.7)	4.0 (2.1)	<0.001
Education (yrs)	11.7 (4.3)	10.7 (3.9)	0.002	12.0 (4.4)	11.8 (4.3)	0.566
SPPB score (0–12)	10.5 (1.6)	7.6 (3.0)	<0.001	10.8 (1.4)	9.9 (1.9)	<0.001
Height (m)	*(n = 714)*	*(n = 181)*	**p [c]**	*(n=509)*	*(n=84)*	**p [c]**
Men	1.72 (0.06)	1.71 (0.07)	0.164	1.73 (0.06)	1.71 (0.05)	0.023
Women	1.59 (0.05)	1.57 (0.06)	0.014	1.59 (0.05)	1.59 (0.05)	0.774
Body mass (kg)						
Men	78.6 (11.4)	83.8 (16.1)	0.011	78.5 (11.3)	83.6 (12.6)	0.029
Women	68.9 (11.7)	74.2 (12.2)	<0.001	67.9 (11.0)	77.0 (14.5)	<0.001
BMI (kg/m^2)						
Men	26.5 (3.6)	28.6 (5.0)	0.001	26.3 (3.4)	28.8 (4.3)	0.002
Women	27.4 (4.4)	30.1 (5.1)	<0.001	26.9 (4.1)	30.5 (5.9)	<0.001
6MWT speed (m/s)	*(n = 703)*	*(n = 151)*		*(n=503)*	*(n=82)*	
Men	1.25 (0.19)	0.90 (0.26)	<0.001	1.28 (0.17)	1.14 (0.19)	<0.001
Women	1.17 (0.20)	0.87 (0.21)	<0.001	1.21 (0.18)	0.99 (0.19)	<0.001
Knee extension strength (N)	*(n = 709)*	*(n = 176)*		*(n=505)*	*(n=84)*	
Men	430 (100)	363 (104)	<0.001	441 (96)	401 (101)	0.033
Women	292 (82)	248 (75)	<0.001	303 (83)	257 (70)	<0.001

Note. SPPB, Short physical performance battery; BMI, Body Mass Index; 6MWT, 6-minute walking test at preferred speed; P-values are calculated with [a] Chi-square test, [b] Mann-Whitney U Test or [c] Independent T-test.

eTable 2. Physical activity from the self-report and accelerometry surveillance at baseline stratified for the prevalence and incidence of advanced (500 m) walking difficulty.

	Prevalence (baseline)			Incidence (2-yr follow-up)		
	No difficulty	Prevalent difficulty		No difficulty	Incident difficulty	
	Mean (SD)	Mean (SD)	p	Mean (SD)	Mean (SD)	p
Self-report	*(n=769)*	*(n=220)*		*n=(533)*	*n=(93)*	
YPAS total score	59 (23)	41 (20)	<0.001	62 (22)	46 (19)	<0.001
YPAS vig+walk (min/week)	267 (145)	160 (117)	<0.001	284 (144)	187 (122)	<0.001
YPAS vig (min/week)	116 (100)	60 (70)	<0.001	123 (99)	78 (85)	<0.001
YPAS walk (min/week)	151 (97)	99 (80)	<0.001	161 (98)	109 (78)	<0.001
PA level (1-6)	3.7 (0.8)	2.8 (0.9)	<0.001	3.8 (0.8)	3.1 (0.8)	<0.001
Accelerometry surveillance	*(n=398)*	*(n=88)*		*(n=299)*	*(n=38)*	
Average acceleration (mg)	25.4 (8.2)	18.0 (6.5)	<0.001	26.2 (8.2)	20.3 (6.3)	<0.001
$MVPA_{abs}$ (min/week)	241 (164)	107 (111)	<0.001	256 (167)	148 (122)	<0.001
PA_{rel} (min/week)	66 (87)	43 (47)	0.002	68 (92)	47 (61)	0.083

Note. YPAS, Yale Physical Activity Survey; PA, physical activity; $MVPA_{abs}$, moderate to vigorous PA based on an absolute accelerometry cut-point; PA_{rel}, PA relative to preferred walking speed; P-values are calculated with Independent T-test.

eMethods. Logistic regression analysis using z-scores

Logistic regression analysis was additionally performed by converting the accelerometry measures to z-scores in a sample with complete data set (n=278). The aim was first, to allow comparison of the odds ratios between the different PA methods and units and second, to remove the effect of the systematically smaller analytic sample in accelerometry compared to self-report (eTable 3).

eTable 3. Logistic regression for the incidence of early (2 km) walking difficulty in the 2-year follow-up using z-scores for physical activity.

	Model 1 unadjusted		Model 2 adjusted		Model 3 fully adjusted	
	OR	[95% CI]	OR	[95% CI]	OR	[95% CI]
Self-report	*(n=278)*		*(n=278)*		*(n=278)*	
YPAS total score (1 SD)	0.62*	[0.43, 0.91]	0.61*	[0.41, 0.91]	0.72	[0.48, 1.09]
YPAS vig+walk (1 SD)	0.60*	[0.40, 0.88]	0.58*	[0.38, 0.89]	0.73	[0.47, 1.13]
YPAS vig (1 SD)	0.67*	[0.46, 0.99]	0.63	[0.46, 1.02]	0.81	[0.54, 1.21]
YPAS walk (1 SD)	0.73	[0.51, 1.05]	0.71	[0.48, 1.05]	0.82	[0.54, 1.24]
PA level (1 SD)	0.60*	[0.43, 0.84]	0.63*	[0.44, 0.90]	0.86	[0.58, 1.27]
Accelerometer surveillance	*(n=278)*		*(n=278)*		*(n=278)*	
Average acceleration (1 SD)	0.44*	[0.27, 0.70]	0.46*	[0.27, 0.75]	0.68	[0.41, 1.12]
$MVPA_{abs}$ (1 SD)	0.43*	[0.26, 0.71]	0.45*	[0.27, 0.75]	0.67	[0.41, 1.10]
PA_{rel} (1 SD)	0.81	[0.54, 1.22]	0.77	[0.50, 1.19]	-	-

Note. OR, odds ratio; CI, confidence interval; YPAS, Yale Physical Activity Survey; vig, vigorous; PA, physical activity; $MVPA_{abs}$, moderate-to-vigorous PA based on absolute accelerometry cut-point; PA_{rel}, PA relative to preferred walking speed. Model 2 is adjusted for age, sex, number of chronic conditions and years of education. Model 3 is additionally adjusted for preferred walking speed in a 6-min walking test. *statistical significance (p<.05).
